# B and T Cell Phenotypic Profiles of African HIV-Infected and HIV-Exposed Uninfected Infants: Associations with Antibody Responses to the Pentavalent Rotavirus Vaccine

**DOI:** 10.3389/fimmu.2017.02002

**Published:** 2018-01-19

**Authors:** Adriana Weinberg, Jane Lindsey, Ronald Bosch, Deborah Persaud, Paul Sato, Anthony Ogwu, Aida Asmelash, Mutsa Bwakura-Dangarambezi, Benjamin H. Chi, Jennifer Canniff, Shahin Lockman, Simani Gaseitsiwe, Sikhulile Moyo, Christiana Elizabeth Smith, Natasha O. Moraka, Myron J. Levin, for the P1072 and Tshipidi Study Teams, Charles Fane

**Affiliations:** ^1^Department of Pediatrics, Section of Pediatric Infectious Diseases, University of Colorado Anschutz Medical Campus, Aurora, CO, United States; ^2^Department of Medicine, Section of Pediatric Infectious Diseases, University of Colorado Anschutz Medical Campus, Aurora, CO, United States; ^3^Department of Pathology, Section of Pediatric Infectious Diseases, University of Colorado Anschutz Medical Campus, Aurora, CO, United States; ^4^Center for Biostatistics in AIDS Research, Harvard School of Public Health, Boston, MA, United States; ^5^W. Harry Feinstone Department of Molecular Microbiology and Immunology, Johns Hopkins Bloomberg School of Public Health, Baltimore, MD, United States; ^6^Maternal Adolescent and Pediatric Research Branch, NIAID, NIH, Bethesda, MD, United States; ^7^Harvard AIDS Institute, Gaborone, Botswana; ^8^Botswana Harvard AIDS Institute, Gaborone, Botswana; ^9^Department of Paediatrics and Child Health, University of Zimbabwe College of Health Sciences, Harare, Zimbabwe; ^10^Department of Obstetrics and Gynecology, University of North Carolina at Chapel Hill, Chapel Hill, NC, United States; ^11^Botswana Harvard AIDS Institute Partnership, Gaborone, Botswana

**Keywords:** human, B cells, T cells, AIDS, antibodies, vaccination

## Abstract

We examined associations between B and T cell phenotypic profiles and antibody responses to the pentavalent rotavirus vaccine (RV5) in perinatally HIV-infected (PHIV) infants on antiretroviral therapy and in HIV-exposed uninfected (PHEU) infants enrolled in International Maternal Pediatric Adolescent AIDS Clinical Trials P1072 study (NCT00880698). Of 17 B and T cell subsets analyzed, PHIV and PHEU differed only in the number of CD4+ T cells and frequency of naive B cells, which were higher in PHEU than in PHIV. In contrast, the B and T cell phenotypic profiles of PHIV and PHEU markedly differed from those of geographically matched contemporary HIV-unexposed infants. The frequency of regulatory T and B cells (Treg, Breg) of PHIV and PHEU displayed two patterns of associations: FOXP3+ CD25+ Treg positively correlated with CD4+ T cell numbers; while TGFβ+ Treg and IL10+ Treg and Breg positively correlated with the frequencies of inflammatory and activated T cells. Moreover, the frequencies of activated and inflammatory T cells of PHIV and PHEU positively correlated with the frequency of immature B cells. Correlations were not affected by HIV status and persisted over time. PHIV and PHEU antibody responses to RV5 positively correlated with CD4+ T cell counts and negatively with the proportion of immature B cells, similarly to what has been previously described in chronic HIV infection. Unique to PHIV and PHEU, anti-RV5 antibodies positively correlated with CD4+/CD8+FOXP3+CD25+% and negatively with CD4+IL10+% Tregs. In conclusion, PHEU shared with PHIV abnormal B and T cell phenotypic profiles. PHIV and PHEU antibody responses to RV5 were modulated by typical HIV-associated immune response modifiers except for the association between CD4+/CD8+FOXP3+CD25+Treg and increased antibody production.

## Introduction

In the absence of antiretroviral therapy (ART), HIV-infected persons maintain high levels of inflammation and increased activation, exhaustion, and immune senescence (1–6). The distribution of many B and T cell subsets tends to normalize after initiation of ART, although not completely (7, 8). Although immune responses to vaccines and infections also improve with ART, their relationship with the reconstitution of B and T cell phenotypic profiles is incompletely understood. In our previous studies, we showed complex interactions between adaptive immune responses to vaccines and T cell activation and regulation (9). For example, humoral and cellular immune responses to inactivated influenza vaccines in HIV-infected children, adolescents and pregnant women were lower in vaccines with higher proportions of nonspecific activated and regulatory T cells (Treg) and exhausted B cells in the peripheral blood (9–11). Conversely, vaccination increased influenza-specific Treg and activated T cells proportional to the increase in effector T cells. Muyanja et al. showed that antibody responses to yellow fever vaccine of HIV-infected and uninfected adults were lower in vaccinees with higher activation markers at the time of vaccination, although their study did not control for the HIV status of the participants (12). We also showed inverse correlations of plasma IFNγ and IL10 levels with neutralizing antibody responses to the pentavalent rotavirus vaccine (RV5) (13). Despite recent advances in understanding factors that modulate immune responses, many questions remain regarding the relationship between B and T cell phenotypic profiles and protective immune responses to vaccines in HIV-infected individuals.

Much less is known about the immune system of perinatally HIV-infected (PHIV) infants who start ART early in life, except that they mount higher antibody responses to vaccines compared with PHIV with delayed therapy (14). There is conflicting information about vaccine responses in HIV-exposed uninfected (PHEU) infants, with some studies showing antibody responses comparable to those of HIV-unexposed children and other studies reporting lower antibody and cell-mediated immune responses (15–21). Both PHIV and PHEU have increased risk of severe infections, hospitalizations and death suggestive of immune deficiency (22–25). Although there are reports that PHEU have decreased CD4+T cells and increased T cell activation compared with their HIV-unexposed age-matched controls, overall there is very little information about B and T cell phenotypic profiles in PHEU and PHIV and how they correlate with their immune responses (26–36). Regardless of HIV infection status, infants have decreased immune responses to vaccines and are more susceptible to infections compared with older children and young adults. Infants also have increased Treg at birth compared with older children and young adults, which may contribute to their decreased responses to vaccines. However, very few studies have addressed this question. Fetuses need tolerogenic Tregs to survive in the maternal environment. The tolerogenic role of Tregs was originally described in autoimmune diseases, for which a paucity of critical natural Tregs leads to excessive anti-self T cell responses and inflammation (37–39). By inhibiting conventional T cell, B cell and antigen presenting cell function, Tregs also play an important role in tolerance and allograft retention (40, 41). Treg differentiation starts in the thymus from CD4+T cells. In addition, inducible Tregs with anti-inflammatory activity can be generated to prevent excessive tissue destruction that may result from vigorous immune responses against infectious agents and other foreign antigens (42–47). The Treg hallmark is the transcription factor FOXP3, which inhibits *Ifng* and *Il2* gene transcription and prevents the T cells from differentiating into conventional T cells (48). There are multiple Treg subsets that express additional markers, some of which are associated with their mechanisms of action, including CD25, which binds IL2 with high affinity making it less available to conventional T cells and B cells; CTLA4, which inhibits expression of the activation markers CD80 and CD86 on antigen presenting cells; CD39 and CD73, ectoenzymes that cooperatively dephosphorylate ATP to adenosine, which is immunotoxic to other mononuclear cells; granzyme B, which induces apoptosis of the cytotoxic Treg targets; galectin-3, which prevents the formation of the immunologic synapses; LAG-3, which binds to MHCII inhibiting MHCII-expressing immune cells; PD-1, which binds to PDL-1 and inhibits conventional T cells and induces tolerogenic antigen presenting cells; TNFRII, which induces apoptosis; and the inhibitory cytokines IL10, IL35 and TGFβ (42).

To start addressing the potential role of Treg and B cells in the decreased immune responses of PHIV and PHEU and the potential interactions between the different T and B cell subsets, which were investigated here *de novo* with the intention of generating new hypotheses, we examined in an exploratory fashion select B and T cell subsets in PHIV and PHEU before and after vaccination with RV5. The parent study was a double-blind placebo-controlled trial that enrolled PHEU and PHIV on or initiating ART (49). The study showed that PHIV and PHEU tolerated RV5 equally well and mounted similar antibody responses. This report addresses additional objectives included in the parent study: (1) to compare T cell activation and regulation and B cell differentiation in PHIV and PHEU; (2) to determine the effect of RV5 administration on B and T cell subsets; and (3) to determine the role of regulatory, activated and inflammatory T cells, and of B cell differentiation, on the antibody response to RV5.

## Participants and Methods

### Study Design

The parent clinical trial (P1072), sponsored by the International Maternal Pediatric Adolescent AIDS Clinical Trials network, was a Phase II randomized, placebo-controlled, double-blind study of RV5 in infants born to HIV-infected mothers in 4 African countries where rotavirus vaccination was not part of the national immunization program (49). Infants between 2 and <15 weeks of age at screening were determined to be PHEU or PHIV. Infants in each stratum were randomized to receive three doses of RV5 or placebo according to the recommended schedule of immunization for RV5. Participants were followed until 6 weeks after the last dose, with visits at 7, 14, 21, and 42 days after each dose, to record clinical signs, symptoms and new significant diagnoses. Blood for immunogenicity, plasma cytokines and lymphocyte phenotypic profiles was collected at entry, 21 days after the first dose of vaccine and 14 and/or 42 days after the third dose.

Samples included in these analyses were obtained from infants who received all three doses of vaccine per-protocol, had sufficient numbers of peripheral blood mononuclear cell (PBMC) for flow cytometry at ≥3 time points and had blood collections performed within allowable time intervals (before the first dose, 14–28 days after the first dose, 11–21 and 28–70 days after the third dose). To ensure roughly equal numbers of PHIV and PHEU, only participants enrolled between February 2009 and January 2013 were included. After January 2013, enrollment mostly consisted of PHEU.

To place the B and T cell subset distribution of PHIV and PHEU in the context of HIV-unexposed hosts, we used a convenience set of cryopreserved PBMC from 6-month infants born to HIV-uninfected mothers who were enrolled in an observational study (“Tshipidi”) in Botswana (50).

### Antibody Measurements

Antibody responses to RV5, measured on serum obtained at baseline and 14 or 42 days after the third dose of vaccine, included serum neutralizing antibodies targeting the viral capsid proteins G1, G2, G3, G4, and P1A, and IgA antibodies that recognize RV5 epitopes in RV5-infected fibroblasts. Antibody responses were expressed in units/mL (49).

### Flow Cytometric Analysis

Peripheral blood mononuclear cells were cryopreserved at clinical site laboratories approved by the DAIDS-sponsored cryopreservation quality assurance program for protocol work. B and T cell subsets were enumerated in freshly thawed cryopreserved PBMC that met previously described testing criteria (51). After washing and counting viable cells, PBMC were surface-stained with the following conjugated mAbs: anti-CD3-AF488 (Biolegend; clone HIT3a), anti-CD8-APC/AF750 (Invitrogen; clone 3B5), anti-CD8-PE/Cy7 (Invitrogen; clone 3B5), anti-CD19-PE/Cy5 (BD Biosciences; clone HIB19), anti-CD19-PE/Cy7 (eBioscience; clone HIB19), anti-CD-10-APC/Cy7 (Biolegend; clone HI10a), anti-CD25-APC/Cy7 (BD Biosciences; clone M-A251), CD21-PE/Cy5 (BD Biosciences; clone B-ly4), CD38-PE (Invitrogen; clone HIT2), CD27-APC (BD Biosciences; clone L128); HLA-DR-PE/Cy5 (BD Biosciences; clone G46-6). Cells were fixed and permeabilized using a Fixation/Permeabilization kit (eBioscience), and stained with IL-10-APC (BD Biosciences; clone JES3-19F1), IL-17-PE/Cy7 (Biolegend; clone BL168), FOXP3-APC (eBioscience; clone PCH101), TGFβ-PE (Cedarlane; clone TB21) and analyzed with Guava easyCyte 8HT and FlowJo (Treestar) in three independent panels containing the following markers: (1) CD3, CD8, CD38, CD25, HLA-DR, and FOXP3; (2) CD3, CD8, CD19, TGFβ, IL-10, and IL-17; (3) CD19, CD10, CD21, and CD27. T and B cell subsets were expressed as percentages of the parent CD4+, CD8+, or CD19+ cell populations. The gating strategy for each of the three independent six-color panels is presented in Figure S1 in Supplementary Material.

### Statistical Analysis

Analyses focused on univariate comparisons as the small sample sizes limited use of multivariate techniques. Correlations with significance levels of *p* < 0.1 were highlighted in the figures and text. The less stringent significance level was used as we did not want to miss signals of potential biological significance. Because of the large number of markers and the exploratory nature of the analyses, results should be viewed as hypothesis-generating.

Distributions of biomarkers at entry were compared by HIV-1 status using Wilcoxon rank sum tests. Spearman correlations were calculated among biomarkers and other participant characteristics. To assess changes in marker levels over time, mixed models with random intercepts and slopes were fit on the log_10_-transformed measurements to determine whether the slopes were associated with HIV-1 status or vaccine administration. To reflect marker level at entry and over time, the area under the curve (AUC) was calculated using individual-level effects from the mixed models, and the AUC correlated with antibody responses to RV5 in vaccine recipients.

## Results

### Demographic and HIV Disease Characteristics

**Figure 1:**
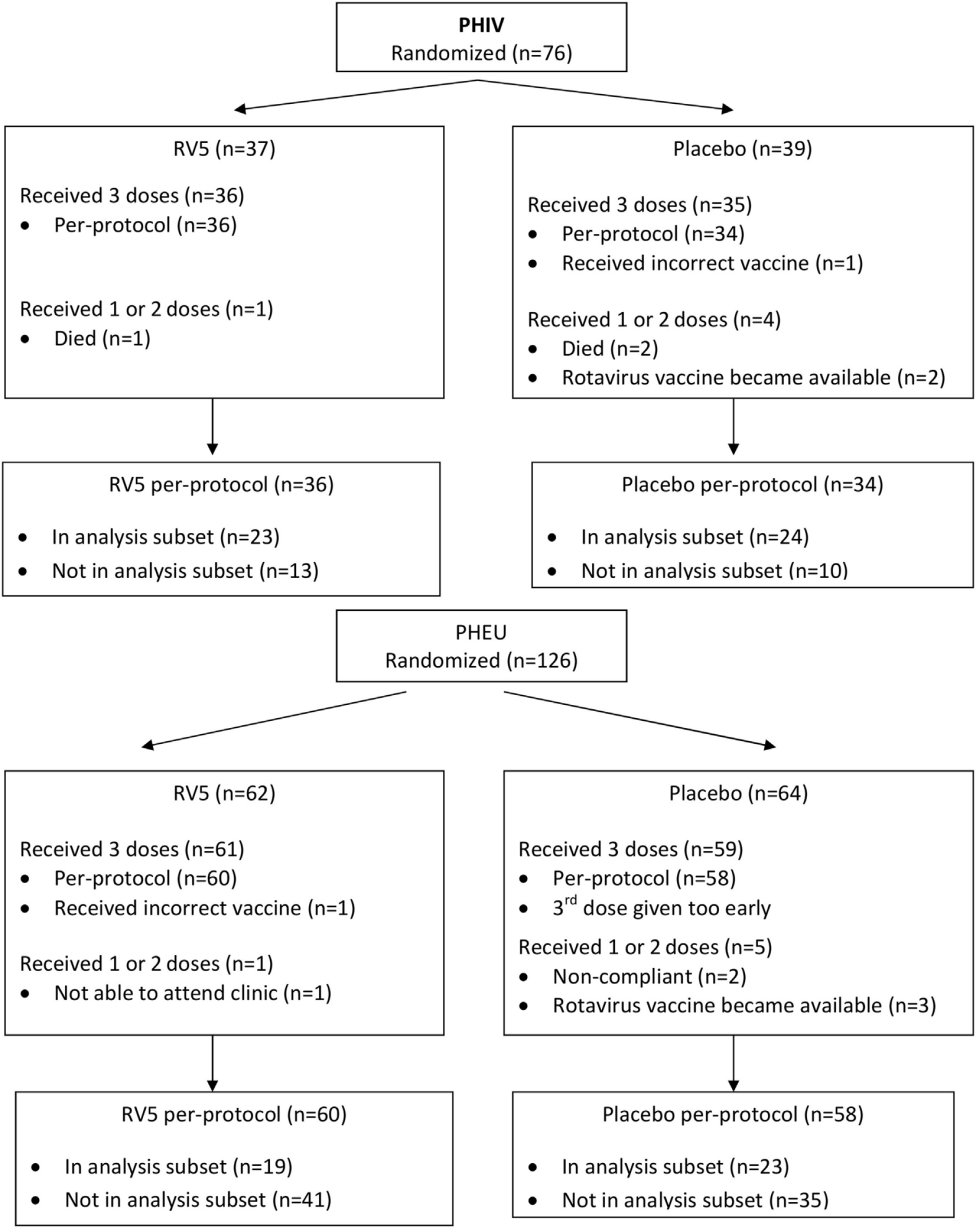
Consort diagram of the parent study.

This analysis included data from 89 of the 202 participants in the parent study (47 of 76 PHIV and 42 of 126 PHEU) who received all three doses of vaccine or placebo per-protocol and had cryopreserved PBMCs at entry, postdose 1 and postdose 3 (Figure 1). Table 1 shows that the demographic characteristics of the participants with flow cytometry data were similar to those who did not contribute PBMC for this analysis. Overall, PHIV and PHEU differed with respect to maternal ART for prevention of mother to child HIV transmission, which was more frequently used in PHEU. Weight *z*-scores were lower in PHIV as were CD4+ T cells, with 15% of PHIV and none of the PHEU displaying CD4+ T cells <20%. PHIV were either on ART at study entry or started ART with the administration of the first dose of vaccine. Median plasma HIV RNA of PHIV at entry was 33,491 cp/mL.

**Table 1:** Characteristics at study entry by HIV status.

Characteristic		PHIV (*N* = 76)	PHIV: with flow (*N* = 47)	PHEU: with flow (*N* = 42)	PHEU (*N* = 126)
Country	Botswana	33 (43%)	17 (36%)	11 (26%)	37 (29%)
	Tanzania	6 (8%)	5 (11%)	5 (12%)	7 (6%)
	Zambia	6 (8%)	3 (6%)	4 (10%)	8 (6%)
	Zimbabwe	31 (41%)	22 (47%)	22 (52%)	74 (59%)

Sex	Male	35 (46%)	20 (43%)	15 (36%)	59 (47%)
	Female	41 (54%)	27 (57%)	27 (64%)	67 (53%)

Age at randomization (days)	Median (min, max)	93 (39, 104)	93 (61, 104)	92 (52, 101)	80 (28, 103)
Ever breast fed	Yes	48 (63%)	30 (64%)	23 (55%)	79 (63%)
	No	28 (37%)	17 (36%)	19 (45%)	47 (37%)

PMTCT	No	25 (33%)	14 (30%)	8 (19%)	13 (10%)
	Yes	51 (67%)	33 (70%)	34 (81%)	113 (90%)

Mother on ARVs	No	62 (82%)	38 (81%)	14 (33%)	49 (39%)
	Yes	14 (18%)	9 (19%)	28 (67%)	77 (61%)

ARV (days)^a^	Median (Min, Max)	4 (0, 50)	6 (0, 49)		
TMP/SMX	No	6 (8%)	3 (6%)	15 (36%)	34 (27%)
	Yes	70 (92%)	44 (94%)	27 (64%)	92 (73%)

WHO weight-for-age *z*-score	Median (Q1, Q3)	−1.5 (−2.4, −0.3)	−1.4 (−2.7, −0.2)	−0.7 (−1.4, −0.1)	−0.6 (−1.3, −0.1)
Screening CD4%	Median (min, max)	29 (7, 58)	28 (7, 58)	36 (20, 66)	38 (19, 66)
	<15%	3 (4%)	3 (6%)	0 (0%)	0 (0%)
	15 to <20%	8 (11%)	4 (9%)	0 (0%)	1 (1%)
	≥20%	65 (86%)	40 (85%)	42 (100%)	125 (99%)

Entry HIV-1 RNA (copies/ml)	Median	48,314	33,491		
	<10 K	22 (31%)	15 (32%)		
	10 to <100 K	18 (25%)	14 (30%)		
	100 to <750 K	15 (21%)	10 (21%)		
	≥750 K	17 (24%)	8 (17%)		
	Not measured	4	0		

Vaccine group	RotaTeq	37 (49%)	23 (49%)	19 (45%)	62 (49%)
	Placebo	39 (51%)	24 (51%)	23 (55%)	64 (51%)

*^a^Six infants were not on ARVs when they received the first study vaccination*.

*PMTCT, prevention of mother-to-child transmission; ARV, antiretroviral therapy; TMP/SMX, cotrimoxazole; PHEU, perinatally HIV-exposed uninfected; PHIV, perinatally HIV-infected infants*.

### Baseline B and T Cell Phenotypic Profiles

B and T cell profiles were identified by flow cytometry in freshly thawed PBMC and presented as proportions of the parent B, CD4+ or CD8+ T cell populations. At entry, there were no statistically significant differences between PHEU and PHIV infants in the proportions of activated and inflammatory T cells, Breg and Treg (Table 2). Compared with PHEU, PHIV had lower proportions of CD19+ CD10−CD21+ CD27− naive B cells (medians of 25.5 vs. 21.4%; *p* = 0.04). The similarity of the B and T cell phenotypic profiles in PHIV and PHEU was unexpected. To interpret these results, we tested a convenience sample of four 6-month old HIV-unexposed infants from a contemporary observational study conducted at our Botswana study site (50). We found significant differences in the proportions of multiple B and T cell subsets of PHIV and PHEU compared with the HIV-uninfected unexposed (HUU) infants, including higher inflammatory CD4+ IL17+ % T cells and lower naive B cells, activated CD4+/CD8+ CD38+ HLADR+ % T cells and IL10+ % Treg and Breg (Table 2).

**Table 2:** B and T cell phenotypic characteristics [median (Q1, Q3) *N*] by HIV exposure.

Assay	Median (Q1, Q3); *N*			*p*-Values		
	PHEU	PHIV	HUU	PHIV:PHEU	PHIV:HUU	PHEU:HUU
CD4+: %CD38+ HLADR+ (activated T cells)	7.1 (5.0, 10.8); 38	7.9 (4.3, 10.8); 39	27.5 (22.9, 39.8); 4	0.87	0.002	0.002
CD4+: %IL17+ (mucosal inflammatory T cells)	2.8 (1.3, 5.9); 40	3.2 (1.6, 6.8); 42	0.7 (0.4, 1.2); 4	0.71	0.013	0.014
CD4+: %CD25+ FOXP3+ (regulatory T cells)	0.1 (0.0, 0.2); 38	0.1 (0.0, 0.2); 39	0.3 (0.2, 0.4); 4	0.43	0.11	0.06
CD4+: %IL10+ (regulatory T cells)	2.3 (1.0, 3.4); 40	2.0 (1.3, 3.2); 42	5.8 (5.2, 7.5); 4	0.71	0.004	0.014
CD4+: %TGFβ+ (regulatory T cells)	2.8 (1.6, 4.3); 40	2.5 (1.5, 5.2); 42	4.5 (2.6, 5.6); 4	0.85	0.51	0.37
CD8+: %CD38+ HLADR+ (activated T cells)	14.4 (10.8, 21.6); 37	13.1 (10.4, 19.3); 38	23.4 (21.9, 39.1); 4	0.62	0.030	0.043
CD8+: %IL17+ (mucosal inflammatory T cells)	4.8 (2.4, 11.1); 41	4.7 (2.2, 10.3); 43	3.3 (1.9, 5.1); 4	0.92	0.35	0.34
CD8+: %CD25+ FOXP3+ (regulatory T cells)	0.3 (0.1, 0.5); 37	0.2 (0.1, 0.4); 38	0.4 (0.3, 0.5); 4	0.21	0.16	0.46
CD8+: %IL10+ (regulatory T cells)	2.3 (1.5, 6.0); 41	1.8 (1.3, 5.6); 43	3.1 (2.0, 4.7); 4	0.66	0.61	0.81
CD8+: %TGFβ+ (regulatory T cells)	3.2 (2.0, 5.6); 41	3.8 (1.7, 8.2); 43	5.5 (2.9, 7.4); 4	0.73	0.70	0.40
CD19+: %CD10+ (immature B cells)	11.3 (6.6, 25.2); 41	11.9 (6.1, 23.9); 42	12.1 (10.1, 15.0); 4	0.89	0.82	0.97
CD19+: %C10− CD21+ CD27− (naive B cells)	25.5 (19.0, 33.3); 41	21.4 (11.5, 27.4); 42	51.4 (45.5, 60.2); 4	0.040	0.004	0.006
CD19+: %C10− CD21+ CD27+ (memory B cells)	32.4 (14.5, 52.5); 41	35.0 (17.3, 49.2); 42	15.4 (10.8, 18.2); 4	0.82	0.07	0.10
CD19+: %C10− CD21− CD27− (exhausted B cells)	31.6 (21.4, 48.9); 41	27.4 (20.7, 51.8); 42	21.9 (20.8, 23.3); 4	0.95	0.14	0.15
CD19+: %IL10+ (regulatory B cells)	2.5 (1.5, 3.3); 41	2.6 (1.7, 3.6); 39	5.3 (4.0, 7.5); 4	0.89	0.013	0.013
CD19+: %TGFβ+ (active B cells)	4.2 (2.0, 14.8); 41	3.7 (1.5, 11.2); 39	10.0 (4.9, 13.9); 4	0.61	0.24	0.52

*Analyses were performed in perinatally HIV-infected infants (PHIV) and perinatally HIV-exposed uninfected (PHEU) data obtained at study entry; HIV-uninfected unexposed (HUU) had only one data set available at 6 months of life. Comparisons with p-values < 0.05 are bolded*.

**Figure 2:**
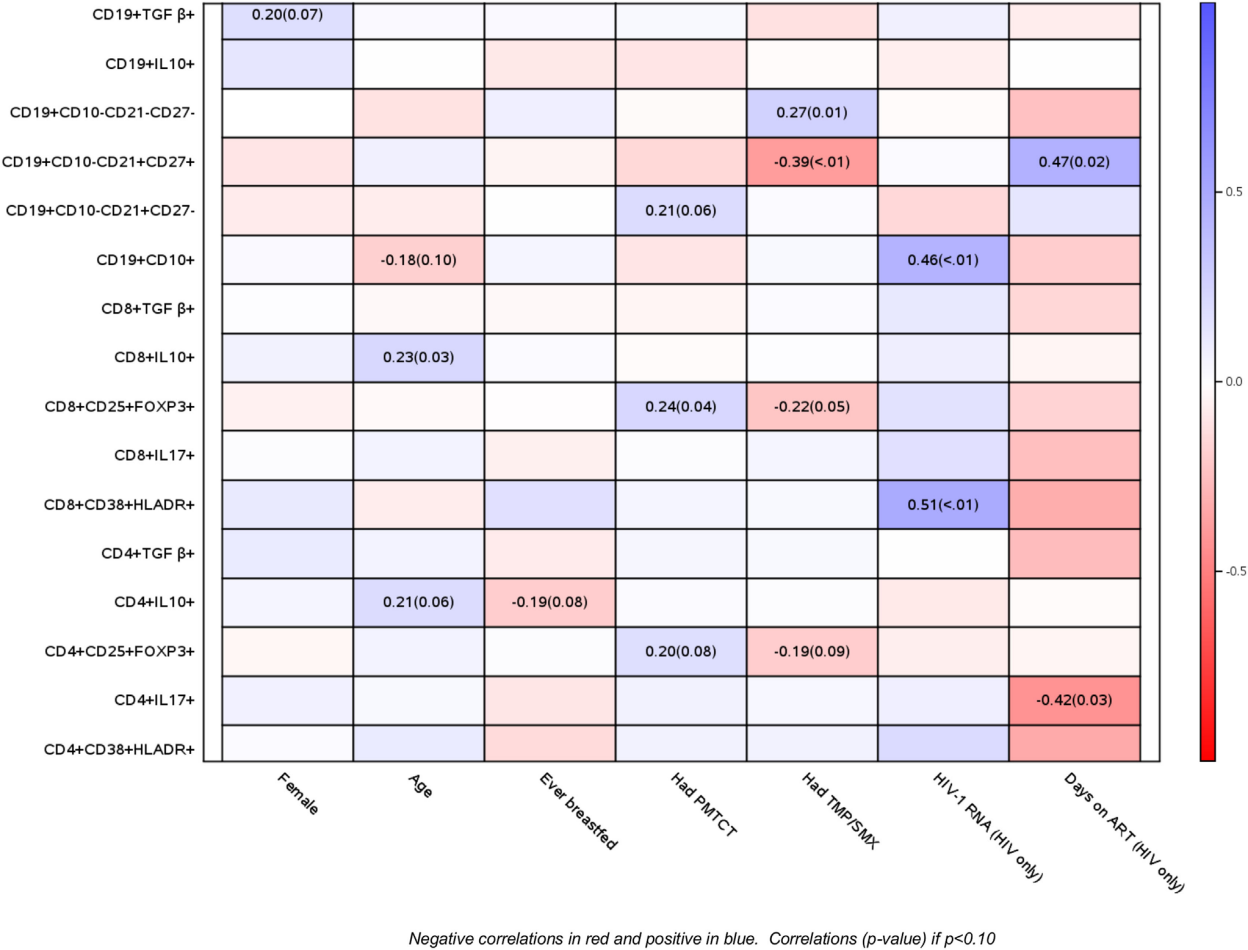
Correlations of B and T cell phenotypic profiles with select demographic and HIV disease characteristics. Data derived from 47 perinatally HIV-infected infants and 42 perinatally HIV-exposed uninfected infants are displayed as a heatmap based on Spearman correlations. Lymphocyte phenotypes are indicated on the *y* axis and demographic and HIV disease characteristics on the *x* axis. Heatmap color legend corresponding to the correlation coefficients is presented on the right side of the graph. The numbers inside the squares indicate coefficients of correlation (*p*-values). Numbers are shown only for correlations with *p* < 0.10.

Using correlation analyses, we further explored the relationship of baseline B and T cell phenotypic profiles of PHIV and PHEU with demographic and HIV disease characteristics (Figure 2). Older age was at least marginally associated with higher CD4+/CD8+ IL10+ % Treg and with lower CD19+ CD10+ % immature B cells. Maternal ART for prevention of mother to child transmission was associated with higher CD4+/CD8+ FOXP3+ CD25+ % Treg and higher naive B cells. Use of cotrimoxazole in infancy was associated with higher exhausted and lower resting memory B cells and marginally associated with lower CD4+/CD8+ FOXP3+ CD25+ % Treg. In PHIV, higher HIV plasma RNA c/mL was strongly associated with higher activated CD8+ CD38+ HLADR+ % and with immature B cells, while the number of days on ART was associated with lower inflammatory CD4+ IL17+ % and higher resting memory B cells. The number of days on ART was also associated with lower activated T cells, but it did not reach statistical significance. There were no differences by gender or breastfeeding status.

### Correlations among the Proportions of B and T Cell Subsets of PHIV and PHEU before and after Vaccination

**Figure 3:**
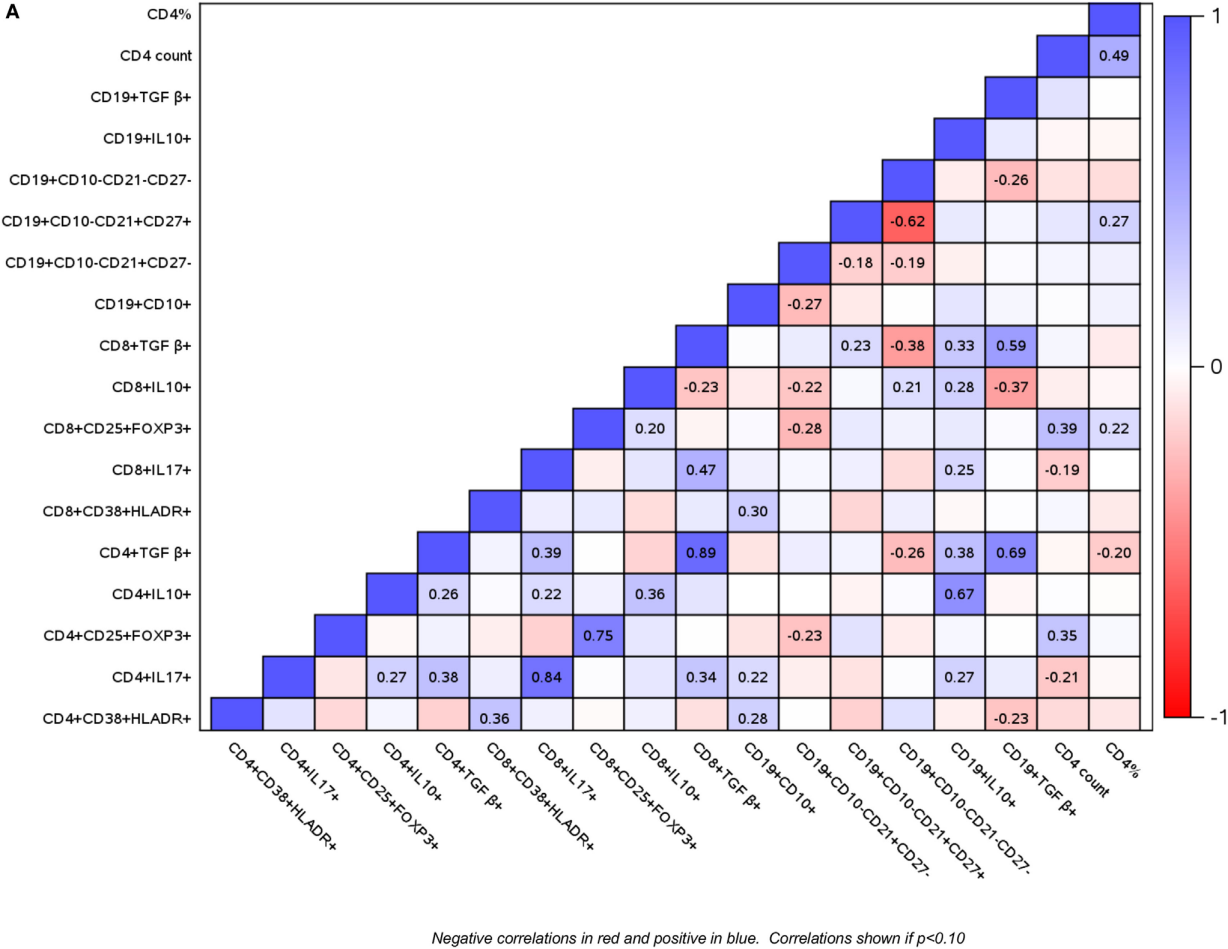

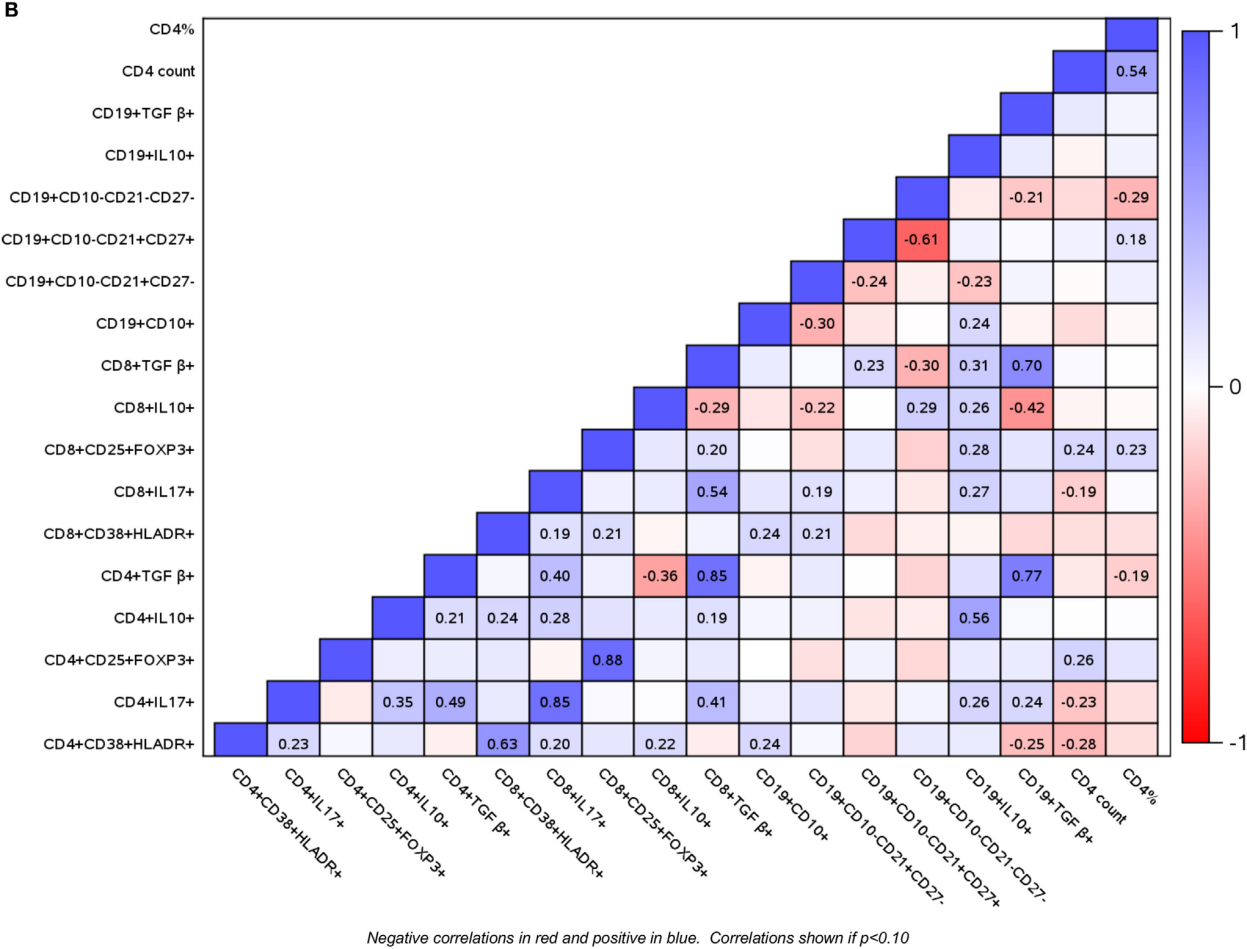
Correlations of B and T cell subsets in perinatally HIV-infected (PHIV) and perinatally HIV-exposed uninfected (PHEU) infants. Data derived from 47 PHIV and 42 PHEU are displayed as a heatmap based on Spearman correlations. Lymphocyte phenotypes are indicated on the *x* and *y* axes. Heatmap color legend corresponding to the correlation coefficients is presented on the right side of the graph. The numbers inside the squares indicate coefficients of correlation. Numbers are shown only for correlations with *p* < 0.10. Panel **(A)** shows data at study entry and panel **(B)** shows data over the entire study period represented by the area under the concentration time curve.

To determine if HIV infection affects the interactions between B and T cell subsets, and if these interactions change over time under the effect of ART or vaccination, we investigated B and T cell subset correlations at entry (Figure 3A) and over time using the AUC as the outcome measure (Figure 3B). In general, the directions of the correlations were similar at baseline and over time, but the magnitude of the coefficients of correlation and p values differed between baseline and over time in some cases. The following is a summary of the most notable correlations. Treg revealed two main clusters of association: (a) high proportions of CD4+/CD8+ FOXP3+ CD25+ Treg positively correlated with CD4+ T cell counts; (b) high proportions of TGFβ+ and IL10+ Treg positively correlated with inflammatory CD4+/CD8+ IL17+ % cells. IL10+ Breg followed the same pattern of associations as the IL10+ Treg. In contrast, CD19+ TGFβ+ % Breg cells correlated only with exhausted B cells. Notable associations of B cells in different stages of maturation included the positive correlations of immature B cells with activated and inflammatory T cells and the negative correlations of immature B cells with mature naive B cells.

### Effect of Vaccination on the Proportions of B and T Cell Subsets

**Figure 4:**
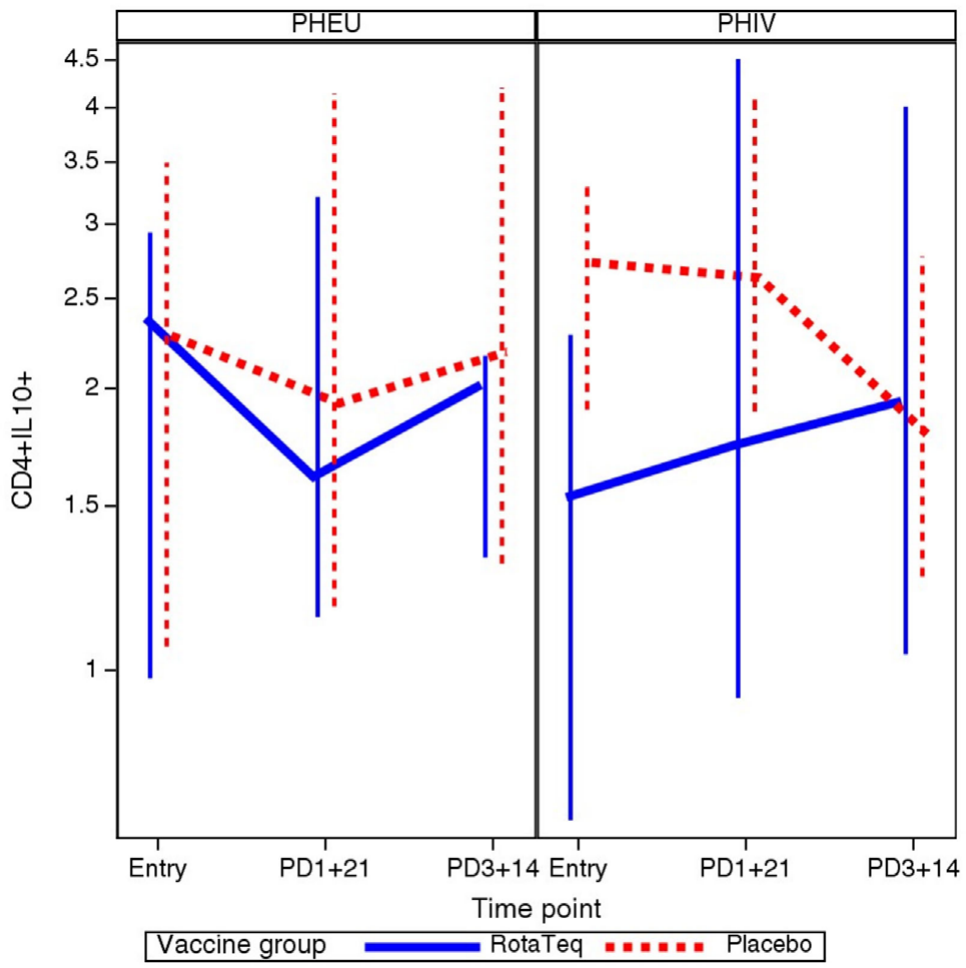
Comparative kinetics of CD4+ IL10+ % in perinatally HIV-infected (PHIV) and perinatally HIV-exposed uninfected (PHEU) who did or did not receive pentavalent rotavirus vaccine (RV5). Data were derived from 19 PHEU who received RV5, 23 PHEU who received placebo, 23 PHIV who received RV5, and 24 PHIV who received placebo. Median CD4+ IL10+ % at each time point indicated on the *x* axis are connected by lines. The bars indicate the lower and upper quartiles. In contrast to all other groups, PHIV vaccinees had an increase of CD4+ IL10+ % Treg after each dose of vaccine (*p* = 0.01 using mixed models analysis).

To determine whether vaccination was associated with a change in the proportions of B and T cell subsets in PHIV or PHEU, we used mixed models to investigate HIV status and treatment group interactions with the slope of each B and T cell marker over time. There was a statistically significant interaction for CD4+ IL10+ % Treg, such that only PHIV vaccine recipients showed an increase in the proportions of this subset after each dose of vaccine (*p* = 0.013; Figure 4). There were no significant interactions of HIV status with treatment group (vaccine or placebo) for any other B and T cell subsets.

### Relationship between the Proportions of B and T Cell Subsets and Antibody Responses to RV5

As we previously reported (49), 81% of the PHIV and PHEU in this study had IgA responses to RV5, defined as ≥3-fold increases from pre- to postvaccination. Interpretation of the IgG neutralizing antibody responses after vaccination was complicated by pre-existing maternal antibodies. Median anti-G1 and G4 IgG neutralizing antibody levels increased from pre- to postimmunization in PHIV and PHEU vaccinees, but not in placebo recipients. Anti-G2, G3 and P1 neutralizing antibodies did not show absolute increases from pre- to postimmunization in vaccinees, but showed decreases in placebo-recipients. Taken together, the data showed higher neutralizing titer differences from pre- to postimmunization in vaccinees compared to placebo-recipients for all RV5 antigens in PHEU, and for G1, G3, G4, and P1 in PHIV (Table S1 in Supplementary Material).

**Figure 5:**
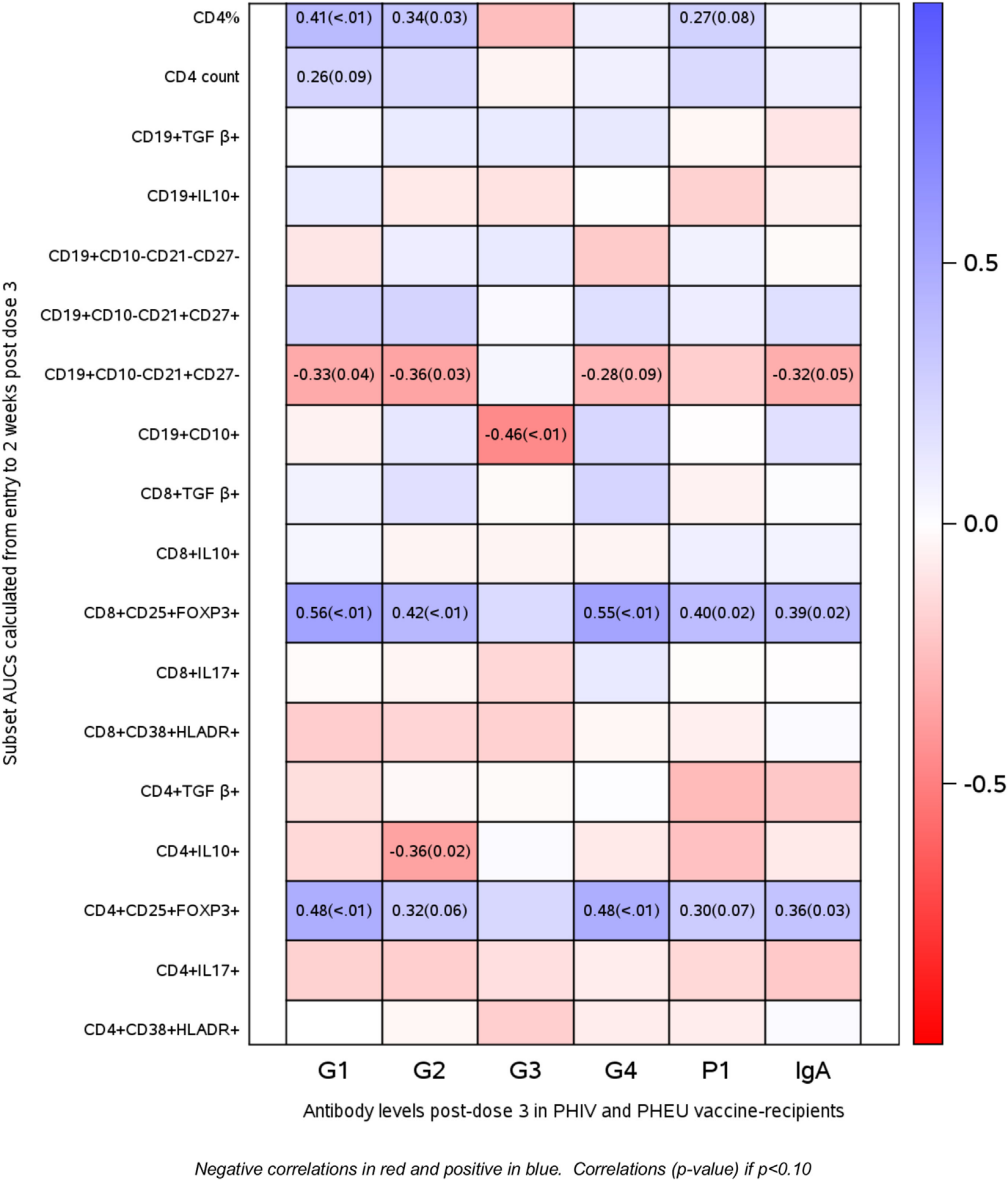
Correlations of antibody responses to rotavirus vaccine (RV5) in perinatally HIV-infected (PHIV) and perinatally HIV-exposed uninfected (PHEU) with T and B cell subsets measured over the duration of the study by AUC. Data derived from 23 PHIV and 19 PHEU who received RV5 are displayed as a heatmap based on Spearman correlations. Lymphocyte phenotypes are indicated on the *y* axis and antibodies to RV5 on the *x* axis. Heatmap color legend corresponding to the correlation coefficients is presented on the right side of the graph. The numbers inside the squares indicate coefficients of correlation (*p*-values). Numbers are shown only for correlations with *p* < 0.10.

Spearman correlations between AUC flow measurements and anti-RV5 antibody levels after the third dose of vaccine in PHIV and PHEU vaccinees (*n* = 42) are presented in Figure 5. The strongest correlations were observed for CD4+/CD8+ FOXP3+ CD25+ %, which were marginally or significantly positively associated with IgG neutralizing antibody responses to four out of five viral strains in RV5 and were significantly positively associated with IgA antibody responses. CD4+ % was marginally or significantly positively associated with higher IgG neutralizing antibody responses to three out of five viral strains in RV5. Correlations of CD4+/CD8+ FOXP3+ CD25+ % Treg with IgG and/or IgA antibody responses to RV5 remained at least marginally significant after adjustment for CD4+ T cell counts or percentages. Negative correlations with antibody titers were noted for CD4+ IL10+ % Treg, immature CD19+ CD10+ % and naive CD19+ CD10− CD21+ CD27− % B cells.

## Discussion

The primary objective of this analysis was to compare the frequency of B and T cell phenotypic subsets of PHIV and PHEU and to identify the phenotypes that correlate with antibody responses to RV5. Contrary to our expectations, PHIV and PHEU had similar proportions of Treg, Breg, activated and inflammatory T cells, and of immature and exhausted B cells in the first 3–6 months of life. Furthermore, the *post hoc* comparison of PHIV and PHEU B and T cell phenotypic profiles with those of a contemporary group of 6-month-old HUU of similar ethnicity and geographic location showed marked differences, indicating that PHIV and PHEU shared immunologic abnormalities as compared with HUU. This is an important observation because PHEU, like the PHIV, have an increased burden of infections compared to HUU, for which the immune basis is poorly understood (52). Our observations provide new information about the immune abnormalities that characterize PHEU and the effect of *in utero* and/or early infancy exposure to maternal HIV infection in shaping the neonate immune system.

IL17 + inflammatory T cells, putatively of mucosal origin, had similar frequencies in PHEU and PHIV and lower frequencies in HUU indicating that PHIV and PHEU might have higher levels of mucosal inflammation. It is important to note that HIV-infected individuals also have higher levels of gut mucosal inflammation compared with healthy hosts (53). Moreover, similar to individuals with chronic HIV infection, PHIV and PHEU had lower proportions of naive B cells and higher proportions of immature and exhausted B cells compared with HUU. However, PHIV and PHEU also had lower activated T cells compared with HUU, which differs from what is typically observed in the context of chronic HIV infection. Although the results of the comparison of HUU with PHIV and PHEU have to be interpreted with caution due to the small number of HUU, our observations suggest that PHIV and PHEU may experience persistent stimulation of B cells and inflammatory T cells that may contribute to their immune deficit.

Treg play an important role in the modulation of immune responses including protection or lack thereof against cancer and infectious agents (43, 54–58). Treg frequencies generally increase in the context of HIV infection. Their role has been alternatively assigned to protect against immune activation or downregulate immune defenses against HIV and other pathogens (59–61). The effect of Treg on vaccine immunogenicity was studied in animal models, where Treg depletion increased the immunogenicity of anti-tumor vaccines (62, 63). Less is known about the effect of Treg on the immunogenicity of vaccines in humans (11, 64). To address this gap, we investigated the association of Treg with the immunogenicity of RV5 in PHIV and PHEU. We found that phenotypically diverse Treg subsets had opposite associations with antibody responses to the vaccine. CD4+ IL10+ % Treg negatively correlated with antibody responses to RV5, whereas the prototypic CD4+/CD8+ FOXP3+ CD25+ % Treg positively correlated with antibody responses. This finding underscores the functional diversity of phenotypically distinct Treg. Treg were previously shown to develop in the thymus or differentiate from naive or conventional T cells at the periphery of the immune system. Treg are also segregated according to their genesis into natural and induced Treg. Additional phenotypic characteristics differentiate Treg according to the targets of their regulatory activity (44, 65). Here we demonstrate that Treg with different phenotypes may have divergent effects on immune responses to a live attenuated vaccine.

The main function of Treg is to mitigate the deleterious effects of inflammation on the host. Consistent with this, we found strong positive correlations of IL10+ and TGFβ+ Treg and IL10+ Breg with inflammatory Th17 cells. Th17 cells typically increase in response to pathogenic signals at mucosal sites, particularly in the gut (66). Collectively these observations suggest that both IL10+ and TGFβ+ Treg may be generated or expanded in PHIV and PHEU in order to quench mucosal inflammation. However, these regulatory responses to inflammation may downregulate other immune responses through a bystander mechanism. This may explain the negative association of CD4+ IL10+ % Treg with IgG antibody responses to G2 in RV5. Although a direct negative effect of IL10 on B cell antibody production is unlikely, since IL10 generally stimulates B cells to secrete antibodies, IL10+ Treg may decrease the T cell help necessary for effective antibody production through a direct effect on conventional T cells and/or indirectly through a tolerogenic effect on antigen presenting cells.

It is important to note that CD4+ IL10+ Treg gradually increased over time in PHIV from pre-vaccination to post-dose 3. The kinetics of CD4+ IL10+ Treg in PHIV vaccine recipients differed from the kinetics of these Treg in the PHEU vaccine recipients or in placebo recipients. The increase in CD4+ IL10+ Treg over time in PHIV vaccine recipients may have contributed to their lower IgG antibody responses to G2 in RV5 compared with PHEU.

In contrast to the IL10+ Treg, high CD4+/CD8+ FOXP3+ CD25+ % Treg were associated with high antibody responses to RV5. This was an unexpected finding, because it differs from previous studies. Our unpublished results and the study of Lelic et al. (64) showed negative associations of CD4+ FOXP3+ CD25+ % with cell-mediated immune responses of older adults to the zoster vaccine, which, like RV5, is a live attenuated vaccine. This difference may be related to the age of the vaccinees, but also to their exposure to maternal HIV infection. Elevated proportions of CD4+/CD8+ FOXP3+ CD25+ % Treg in infants may have a different connotation compared with adults. Fetuses use their CD4+/CD8+ FOXP3+ CD25+ % Treg to survive the allogeneic maternal environment. This property seems to extend to the neonatal period, when T cells continue to differentiate into FOXP3+ CD25+ Treg in response to alloantigens (67). It has been hypothesized that this predominant Treg response contributes to decreased immunogenicity of vaccines in neonates born to healthy mothers (68). However, in PHEU and PHIV the FOXP3+ CD25+ Treg may protect the B cells and/or T helper cells against intense activation followed by apoptosis and allow them to preserve function. In support of this hypothesis was the positive association of the FOXP3+ CD25+ Treg with CD4+ T cell counts and percentages in PHEU and PHIV. Positive associations of FOXP3+ CD25+ Treg with CD4+ T cells were also described in the context of chronic HIV infection and were ascribed to the anti-inflammatory effect of the FOXP3+ CD25+ Treg (61).

We found significant correlations between high CD4+ % T cells and counts and higher IgG antibody responses. This is in agreement with previous studies in HIV-infected individuals whose responses to vaccines increased with higher CD4+ T cells (69, 70).

Similar to what has been previously described in individuals with chronic HIV infection (71), we found a negative association between CD19+ CD10+ % immature B cells and antibody responses to RV5. We also found a negative association of the CD19+ CD21+ CD27− % naive B cells with antibody responses to RV5, which suggests that an impediment of B cell differentiation might underpin lower antibody production.

Our study was limited by the small sample size and by the lack of existing data that might have allowed us to formulate *a priori* hypotheses, The small sample size did not allow us to conduct any type of multivariate analyses. For this reason, instead of using a formal factor analyses, we used the results of the correlation analyses to group the different variables based on our expert knowledge in the discussion. It is also important to note that we achieved our goal of generating new hypotheses.

In conclusion, our data show for the first time that PHIV and PHEU share many aspects previously described in the immune dysfunction that accompanies chronic HIV infection, including increased inflammatory T cells, B cell differentiation defects and immunologic factors that modulate responses to vaccines. We also showed that select B and T cell subsets correlate with antibody responses to RV5 indicating that the abnormal phenotypic profiles of PHEU and PHIV may be functionally relevant. The defects of the PHIV and PHEU immune systems may also underlie the increased infectious morbidity and mortality of these children. Since this study was hypothesis-generating, our findings need to be confirmed and expanded including larger cohorts and geographically distinct populations, in order to achieve a full understanding of the scope of the immune defects in PHEU and design interventions to increase their immune protection.

## Ethics Statement

This study was carried out in accordance with the recommendations of the National Institute of Allergy and Infectious Diseases Division of AIDS and the National Institute of Child Health and Human Development with written informed consent from all subjects. All subjects gave written informed consent in accordance with the Declaration of Helsinki. The protocol was approved by the National IRBs of Botswana, Zimbabwe, Tanzania, and by the Colorado Multiple Institutional Review Board.

## Author Contributions

AW was involved in the study design, overseeing laboratory execution, data analysis and writing of the manuscript. JL and RB participated in data analysis and writing of the manuscript. PS and MJL were involved in study design and reviewing the manuscript. DP and CS contributed to data acquisition and manuscript review. JC participated in laboratory data acquisition and writing manuscript. SG, SM, and NM participated in laboratory data acquisition and reviewing manuscript. AO, AA, MB-D, BC, and SL enrolled subjects and reviewed the manuscript.

## Conflict of Interest Statement

AW: research support, grants, contracts from Merck. ML: consultant, honoraria, royalties, research support, grants, contracts from Merck.
