# Supplementary material for: B and T Cell Phenotypic Profiles of African HIV-Infected and HIV-Exposed Uninfected Infants: Associations with Antibody Responses to the Pentavalent Rotavirus Vaccine
Source: Front Immunol. 2018 Jan 19;8:2002. doi: 10.3389/fimmu.2017.02002 (PMC5780413; doi:10.3389/fimmu.2017.02002)
Supplement: Supplementary file 1 [file Data_Sheet_1.PDF]

Supplementary information

Supplementary Table 1. Median (10th, 90th percentiles) rotavirus antibody levels at entry and post study dose 3[a]

Immunologic assay	HIV status	Time point	Placebo N	Placebo Median	Placebo 10th	Placebo 90th	RV5[b] N	RV5[b] Median	RV5[b] 10th	RV5[b] 90th	Wilcoxon rank sum test: HIV+:HEU (All at entry)[c]	Wilcoxon rank sum test: HIV+:HEU (RV5)[d]	Wilcoxon rank sum test: RV5:Placebo (within HIV- 1 strata)[e]
Serum anti-rotavirus IgA													
	HEU	Entry	58	1	1	25	59	2	1	35	0.08	0.55	
		PD3	58	3	1	257	58	66	5	1137			<0.001
	HIV+	Entry	33	2	1	86	35	2	1	127			
		PD3	33	2	1	267	35	119	7	625			<0.001
Serum Neutralizing Antibody													
SNA G1	HEU	Entry	58	38	13	154	59	42	15	130	0.001	0.92	
		PD3	58	18	10	48	58	53	14	452			<0.001
	HIV+	Entry	33	24	12	91	35	25	12	95			
		PD3	33	15	10	28	35	61	14	324			<0.001
SNA G2	HEU	Entry	58	48	16	139	59	62	21	135	<0.001	0.33	
		PD3	58	21	10	105	58	35	11	167			0.015
	HIV+	Entry	33	29	11	100	35	25	10	118			
		PD3	33	23	10	73	35	23	10	749			0.35
SNA G3	HEU	Entry	58	17	10	200	59	28	10	164	<0.001	0.33	
		PD3	58	10	10	40	58	22	10	159			<0.001
	HIV+	Entry	33	13	10	45	35	12	10	40			
		PD3	33	10	10	12	35	15	10	105			<0.001
SNA G4	HEU	Entry	58	55	19	152	59	67	16	178	<0.001	0.65	
		PD3	58	24	12	101	58	84	26	297			<0.001
	HIV+	Entry	33	27	12	82	35	17	10	56			
		PD3	33	15	10	62	35	101	15	464			<0.001
SNA P1	HEU	Entry	58	67	13	459	59	87	13	311	<0.001	0.11	
		PD3	58	25	10	263	58	60	16	539			0.003
	HIV+	Entry	33	31	10	200	35	32	11	182			
		PD3	33	12	10	145	35	34	10	645			0.015

Lower and upper limits for serum anti-rotavirus IgA: 1.152 and 1250 (dilution=20).
Lower and upper limits for each serum neutralizing antibody (SNA): 10 and 1280 (dilution=2).
Lower limit for rotavirus IgA copro-antibody assay: 0.35 units/ml.
Results were similar when the five participants with pre-dose values greater than 1/3ULOQ were classified as non-responders and when they were classified as responders and included in the analysis.

[a] Per protocol population.
[b] RV5 = pentavalent rotavirus vaccine.
[c] Comparison of pre-entry levels by HIV status.
[d] Comparison of postdose 3 levels by HIV status in RV5 recipients.
[e] Comparison of postdose 3 levels within HIV stratum between RV5 and placebo.

Supplementary Figure 1 (gating strategies)

A. Gating strategy for panels 1 and 2.

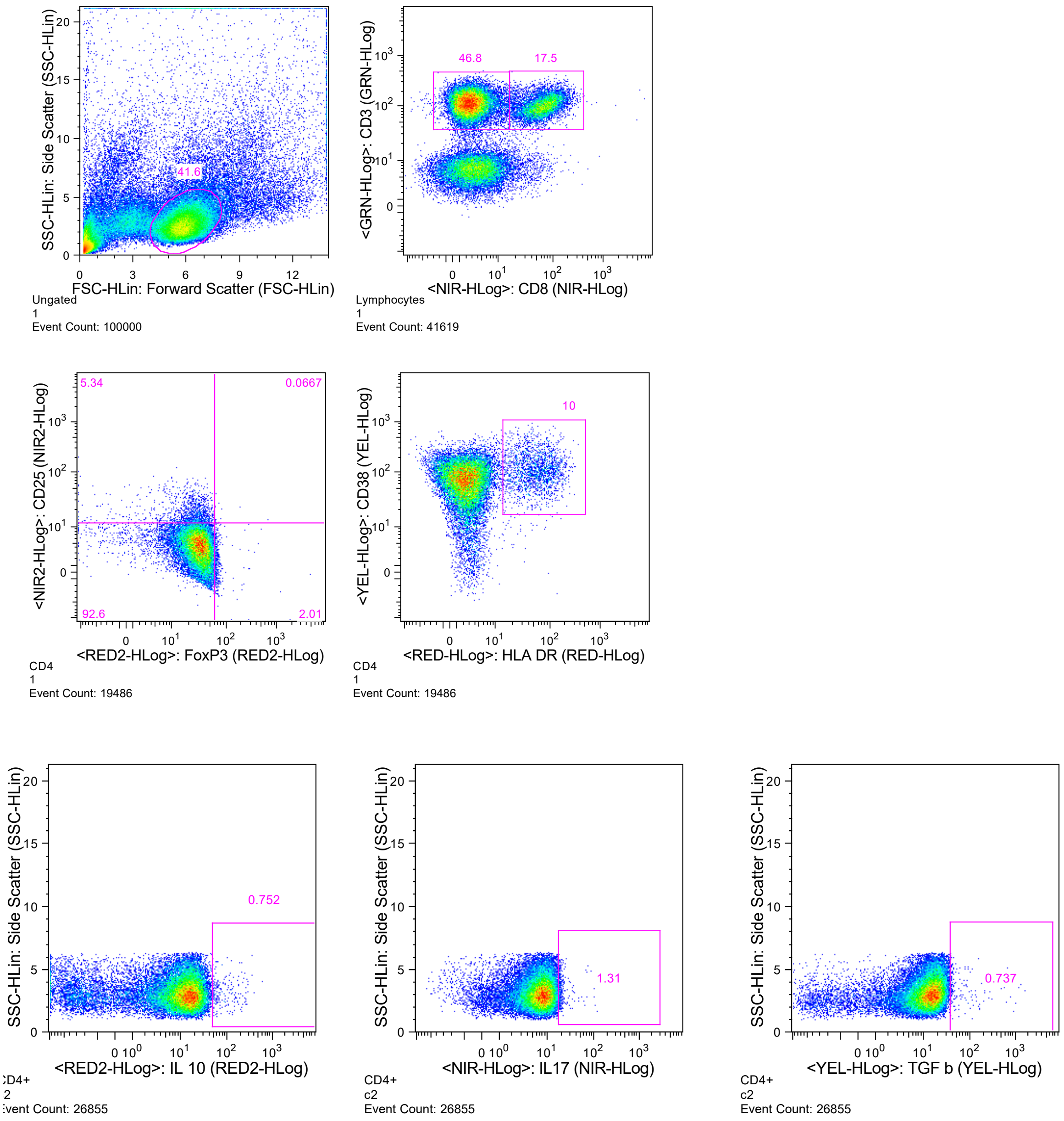

Upper panels show the gating on CD4+ and CD8+ T cells.

Middle panels show an example of gating for panel 1, which included CD3, CD8, HLADR, CD38, CD25 and FOXP3. The panels show gating on CD38+HLADR+ CD8 cells. Similar strategy was used for CD4 cells and for FOXP3 and CD25 both on CD4 and CD8 cells.

Lower panels show an example of gating for panel 2, which included CD3, CD8, CD19, IL10, TGFb and IL17. Due to the small number of events in the TGFb and IL-10 gates, the TGFb+IL10+ population was not evaluated in our comparisons.

B. Gating strategy for panel 3

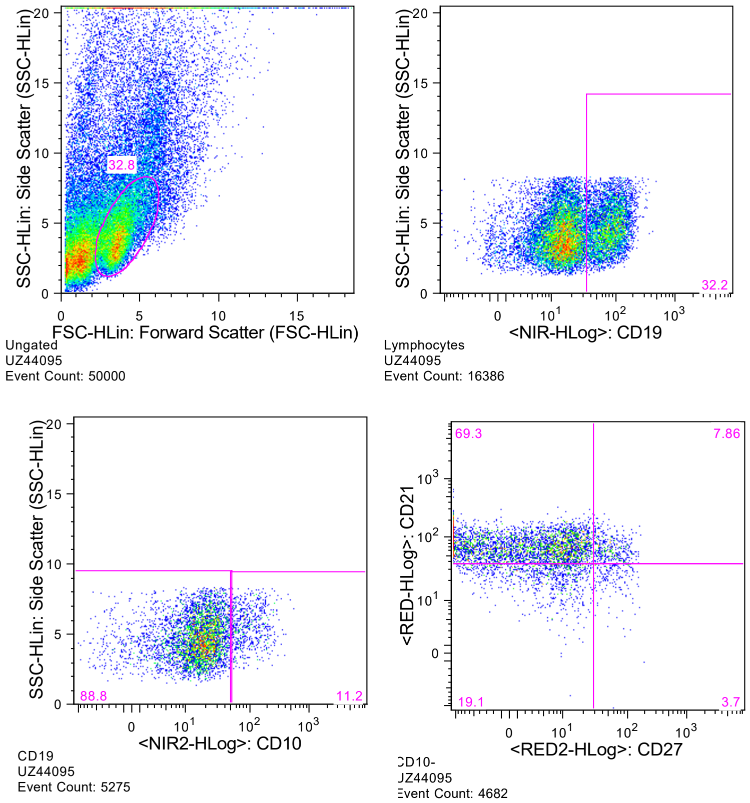

Upper panels show gating the CD19+ B cells

Lower left panel shows gating on CD10+ (left). Lower right shows gating on CD21 and CD27 pos and neg populations out of the CD19+CD10- B cells.
